# Use of single molecule sequencing for comparative genomics of an environmental and a clinical isolate of *Clostridium difficile* ribotype 078

**DOI:** 10.1186/s12864-016-3346-2

**Published:** 2016-12-13

**Authors:** Katherine R. Hargreaves, Anisha M. Thanki, Bethany R. Jose, Marco R. Oggioni, Martha R. J. Clokie

**Affiliations:** 1 Department Infection, Immunity and Inflammation, University of Leicester, Leicester, UK; 2 Department Microbiology, The Ohio State University, Columbus, OH USA; 3 Department Genetics, University of Leicester, Leicester, UK

**Keywords:** Pathogen genomes, SMRT sequencing, Mobile genetic elements, Methlyome, *Clostridium difficile*, Lysogeny, Prophage

## Abstract

**Background:**

How the pathogen *Clostridium difficile* might survive, evolve and be transferred between reservoirs within the natural environment is poorly understood. Some ribotypes are found both in clinical and environmental settings. Whether these strains are distinct from each another and evolve in the specific environments is not established. The possession of a highly mobile genome has contributed to the genetic diversity and ongoing evolution of *C. difficile*. Interpretations of genetic diversity have been limited by fragmented assemblies resulting from short-read length sequencing approaches and by a limited understanding of epigenetic regulation of diversity. To address this, single molecule real time (SMRT) sequencing was used in this study as it produces high quality genome sequences, with resolution of repeat regions (including those found in mobile elements) and can generate data to determine methylation modifications across the sequence (the methylome).

**Results:**

Chromosomal rearrangements and ribosomal operon duplications were observed in both genomes. The rearrangements occurred at insertion sites within two mobile genetic elements (MGEs), Tn*6164* and Tn*6293*, present only in the M120 and CD105HS27 genomes, respectively. The gene content of these two transposons differ considerably which could impact upon horizontal gene transfer; differences include CDSs encoding methylases and a conjugative prophage only in Tn*6164*. To investigate mechanisms which could affect MGE transfer, the methylome, restriction modification (RM)  and the CRISPR/Cas systems were characterised for each strain. Notably, the environmental isolate, CD105HS27, does not share a consensus motif for ^m4^C methylation, but has one additional spacer  when compared to the clinical isolate M120.

**Conclusions:**

These findings show key differences between the two strains in terms of their genetic capacity for MGE transfer. The carriage of horizontally transferred genes appear to have genome wide effects based on two different methylation patterns. The CRISPR/Cas system appears active although perhaps slow to evolve. Data suggests that both mechanisms are functional and impact upon horizontal gene transfer and genome evolution within *C. difficile*.

**Electronic supplementary material:**

The online version of this article (doi:10.1186/s12864-016-3346-2) contains supplementary material, which is available to authorized users.

## Background

*Clostridium difficile* (reclassified as *Clostrioides difficile* [1]) is an enteric pathogenic bacterium that can cause symptomatic disease, which ranges in severity from fever and diarrhoea to the development of pseudomembranous colitis and toxic megacolon [2]. *Clostridium difficile* infection (CDI) occurs following antibiotic treatment as new ecological niches become available upon disruption of the normal microbiota [3]. CDI may arise from ingested endospores transmitted via the faecal oral route, or from vegetative cells already present in the patient, as the bacterium can be asymptomatically carried in adults and children [4]. CDI may also be contracted outside the hospital setting [4], and *C. difficile* has been isolated from food products [4–6], on surfaces around the home [7] and from swimming pools [7]. It has also been isolated from the natural environment including river water, soils, sea water and estuarine sediments [7–10]. The presence of *C. difficile* at these sites may be due to contamination with sewage or agricultural run-off, yet bacteria from these locations could be re-introduced to the food chain, for example via contaminated shellfish or seafood [11, 12], and they have been implicated in the infection of marine mammals [13].

The movement of *C. difficile* between reservoirs is particularly pertinent for isolates of the PCR ribotype 078 (R078). This is an epidemic strain, first identified in livestock and subsequently in clinics across Europe [14]. Although pathogenic, it is not clear quite how much virulence versus strain fitness shapes which strains come to prominence in the hospital environment [15, 16]. R078 strains form a lineage divergent from other major ribotypes [17], as also determined via multilocus sequence typing (MLST) analysis [18, 19] and core genome phylogenies [20, 21]. Previously, we isolated a R078 strain, CD105HS27, from estuarine sediment [9] and sequenced its genome using Illumina HiSeq 2000 generating a draft assembly [22]. The carriage of transposon Tn*6293* (previously unnamed) and the absence of Tn*6164* was confirmed in this study from the results of the Single Molecular Real Time (SMRT) sequencing. The accessory gene content in *C. difficile* as a species is high relative to the size of its core genome [23], and it is characterised by multiple mobile genetic elements which include transposons, integrated conjugative elements, plasmids and prophages (for recent reviews see [23–25]). The acquisition of antibiotic resistance and novel virulence factors are thought to drive *C. difficile* strain pathogen evolution [26], but its ecology outside of the human host is little understood.

Recently, SMRT technology has been applied to sequence *C. difficile* genomes, exploiting the long read data to determine chromosomal structure, mobile genetic content and methylation patterns [27–31]. The re-sequencing of previously analysed strain CD630 showed differences in its ribosomal operon, transposon and tRNA content [28, 31]. In this study we first determined if re-sequencing the reference strain M120 (R078) using SMRT would reveal differences in the chromosomal architecture. Next, we compared SMRT generated genome sequences of M120 with CD105HS27 in order to gain a better understanding of the differences between an environmental isolate and a clinical strain. To date, SMRT has not been applied to isolates of R078. In addition to analysing the genomic data, we compared methylation patterns across the genome. Due to the fact that the CRISPR/Cas system also can provide immunity to invading DNA elements, we assessed its potential to target MGEs for each strain. In both cases, understanding mechanisms that govern horizontal gene transfer in *C. difficile* provides insight into the genome evolution of this pathogen.

## Results and discussion

### Genome features of M120 and CD105HS27

The two genome assemblies generated using SMRT are in near-complete condition; the genome of M120 is 4,082,634 bp with an average coverage of 16.3×, an average 28.73% GC content, and is comprised of two contigs of 4,069,609 bp and 13,024 bp in length. The total sequence for CD105HS27 is 4,122,476 bp, with an overall coverage of 15.75× and an average 29.15% GC content, and consists of five contigs of 3,462,540 bp, 339,877 bp, 174,028 bp, 146,675 bp and 1156 bp, respectively.

**Fig. 1:**
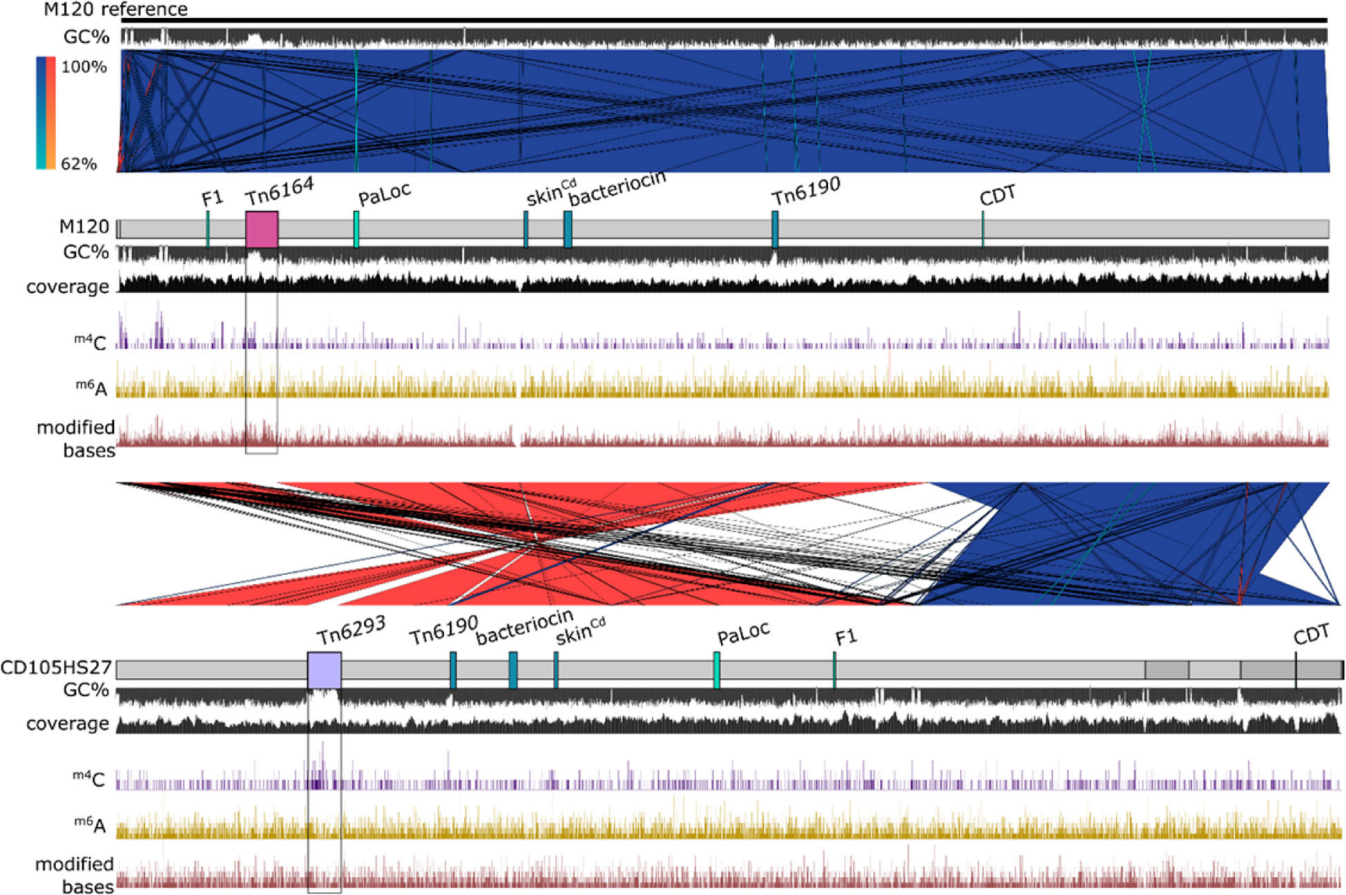
Genome features and comparisons of M120 and CD105HS27. Comparison between M120 reference genome (*top*), M120 sequenced with SMRT (*middle*) and CD105HS27 (*below*). The genomes are connected by regions indicating nucleotide (nt) sequence similarity with notable genomic features annotated at locations along the genome including the PaLoc (Pathogenicity Locus), *C. difficile* binary toxin (CDT) genes, *C. difficile sigK* intervening (skin^Cd^) element, flagella gene region 1 (F1) and annotated transposons. The GC% is provided for all three genomes alongside the coverage and methylation modifications for N4-methylcytosine (^m4^C), N6-methyladenine (^m6^A) and undetermined modified bases. *Boxes* highlight the different methylation patterns observed across each of the unique transposons

Both assemblies were compared to the reference genome of M120, which is a single chromosome 4,047,729 bp in length with an average 28.76% GC content. The 13,024 bp size contig contains a set of 5S, 16S and 23S rRNA genes and 19 tRNA genes, and has duplicated region encoding an identical tRNA (Alanine) and 16S rRNA gene (dot plot data not shown), in addition to predicted CDSs encoding glycosyl transferases, DNA polymerase subunit and recombination protein RecR. The relative coverage of this contig is on average 1.3× (see Fig. 1). To determine whether this contig represents a sequence mobilization event and a low copy number requires experimental investigation.

Annotation of the re-sequenced M120 genome identified 3541 CDSs, 101 tRNAs and 39 rRNAs; this is consistent with the reference genome, but includes an additional 15 tRNAs and 7 rRNA genes. Similar observations were seen  in a SMRT sequenced genome of CD630Δerm with additional tRNA and rRNA genes located in a novel ~5 kbp insertion [28]. This was attributed to adaption during laboratory culture as extra ribosomal gene operon copies have been shown to affect fitness in *E. coli* with regards to nutrient availability [32]. Furthermore, recombination events have been suggested as a mechanism for generating the diversity of ribotypes in *C. difficile* [33].

The genome of CD105HS27 has 3598 CDSs, 93 tRNA and 47 rRNA genes. The chromosome breaks are located in regions encoding ribosomal genes, which appear to have undergone duplication events across the genome. The application of SMRT can also improve the assembly of other regions containing repeat sequences. For example, previously, toxin gene carriage had been confirmed by PCR for CD105HS27 [9], but an Illumina generated draft genome assembly of its genome resulted in fragmented versions of *tcdA* and *tcdB* [22]. Here, these genes have been resolved fully. CD105H27 has 79 CDSs that are not present in M120, most of which are encoded on Tn*6293*, In contrast, M120 has 103 CDSs that are not present in CD105HS27, of which 102 are encoded on Tn*6164*. The predicted genetic content of these two transposons suggests that they may be conjugative transposons although this has yet to be demonstrated experimentally. Therefore, these should be re-termed as putative conjugative transposons CTn*6164* and CTn*6293*. Tn*6164* is a large (~100 kbp) element that appears to be two MGEs including a prophage region which shares similarity to the *Streptoccocus* conjugative phage Φ1207. 3 [34]. Φ1207. 3 has been demonstrated to transfer between strains via conjugation and was originally annotated as a conjugative transposon [35] but contains conserved phage genes including those predicted to encode terminases, capsid, tail and holin proteins leading to its re-designation as a conjugative prophage [36]. Prophages transmitting via conjugation appear rarely in the literature (e.g. [37]). Whether these prophages also transfer via conjugation has not been established, however their discovery suggests that this mechanism may occur more widely than previously known.

**Fig. 2:**
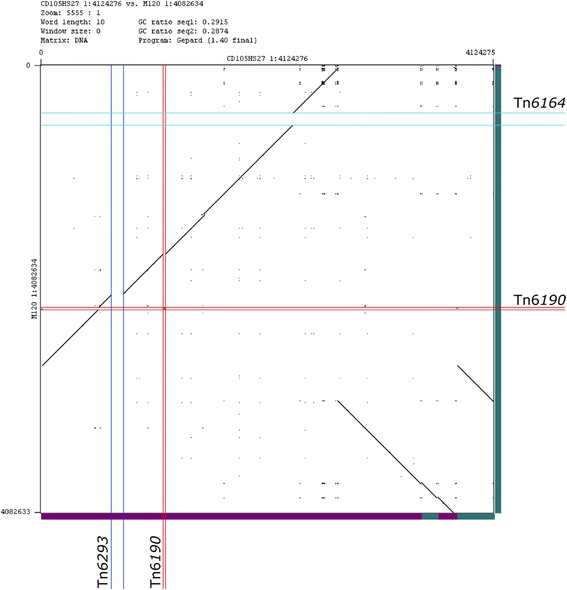
Dotplot of the two genome sequences with indel regions and chromosomal rearrangements. Pairwise comparison of the two nucleotide sequences was performed using a dotplot matrix. The results show regions of shared sequence along the chromosomes (*black line*) and where there are insertion-deletion (indel) events that result in no sequence similarity shared between the genomes (*white gap*). The two largest gaps (~100 kbp each) correspond to the positions of the putative CTns, Tn*6164* in M120 and Tn*6123* in CD105HS27. The conserved but differently positioned Tn*6190* is shown also. The contigs for each genome are illustrated along the sides for each genome to show the chromosomal rearrangements occur within the assembled contig boundaries

The two genomes are related, sharing an average nucleotide identity of 99.98% based on the whole genome sequence (following the method described in [38]). Alignment of the whole genomes using MAUVE and its SNP (Single Nucleotide Polymorphism) detection tool showed that the aligned sequences differed in 85 positions by single nucleotide changes. Further comparison of the two genomes via BLASTn (Fig. 1) and within a dotplot (Fig. 2) revealed extensive sequence similarity between the two strains, with exceptions of two large indel (insertion-deletion) regions (~100 kbp) that carry the putative CTns Tn*6164* and Tn*6123*, the movement of Tn*6190*, and inversion rearrangements. Use of SMRT has previously shown major chromosomal rearrangements from resequencing the genome of strain CD630 in addition to duplication of ribosomal gene operons [28, 30]. One mechanism for these rearrangements are the movement of the MGEs, as seen in the mutant CD630Δerm, where the re-mobilisation of transposon CTn*5* led to the inversion of the genome sequence [28]. What affect such chromosomal re-engineering has on the physiology of the cell in terms of gene expression is not known, but may be significant as has been described for the control of DNA elements from the chromosome in the regulation of diverse bacterial processes [39].

### *In silico* typing of M120 and CD105HS27

In *C. difficile*, ribotyping is one of the main methods used to categorise strains. *In silico* ribotyping was performed to assess the outcomes from the SMRT generated genomes and to explain how the duplication events affect the ribotypes profile. As expected from the different numbers of total rRNA genes, the two profiles differ, with 11 bands predicted from M120 reference, 12 from M120 SMRT and 16 from CD105HS27 (Additional file 1: Table S1). The profiles differ by duplication of identical sized regions in addition to bands of different lengths which may affect ribotypes assigned. While ribosomal gene regions assemble poorly in Illumina datasets, the ability to generate near complete genomes using SMRT technology show how ribosome operon duplication and recombination events could be tracked.

Another method used to type *C. difficile* is MLST (multilocus sequence typing), a scheme that compares the sequence data for seven conserved genes [40]. The two isolate genomes were assigned to Sequence Type (ST) 11, clade 5, which is consistent with previously typed isolates of R078 [19, 40, 41]. The *C. difficile* MLST tool also analysed additional key genes, such as toxins Toxin A, Toxin B and the CDT and also genes that encode for antibiotic resistance. The results confirmed both M120 and CD105HS27 have wild type toxin genes *cdtAB* and *tcdB* and a 39 bp deletion in *tcdC* which has been characteristic of R078 isolates from its early identification [14]. Furthermore, *tetM*, predicted to encode a ribosomal protection protein (CDM120_RS02595) carried on Tn*6190* in M120 [34], is absent in CD105HS27, which has two copies of a variant *tetM*, that share 67% identity at the aa level to that in M120.

### Mobile genetic element content of M120 and CD105HS27

Like other isolates, those from R078 have been found to carry different sets of MGEs which encode for predicted virulence factors and antibiotic resistance [24, 25]. These include the conjugative transposons related to those in other strains of *C. difficile*; Tn*6073* (CTn*1*-like), Tn*6107* (CTn*5*-like) and CTn*4* in the clinical R078 strain QCD-23 M63 [42], as well as those more distantly related, such as Tn*6164* in the reference strain M120 [34]. Tn*6164* is a composite MGE containing a prophage and has several regions that originate from different bacterial lineages [34]. This is considered likely to be a transposon as it can excise and circularise, and carries genes encoding products predicted to be involved in conjugation [34]. While Tn*6164* is characteristically associated with R078 strains, not all R078 isolates carry it [34]. R078 isolates also may harbour Tn*6190* (previously termed CTnCD3a [20]), a Tn*916*-related element that carries the tetracycline resistance gene *tetM* [42], as well as Tn*6235* which carries *aphA1*, an aminoglycoside 3′-phosphotransferase suggested to confer streptomycin resistance [19]. M120 and CD105HS27 both have Tn*6190*, but, as described previously, M120 has Tn*6194* whereas CD105HS27 does not. However, the environmental isolate does have a different large ~104 kbp element [22], now assigned as Tn*6293*. Encoded on Tn*6293* are several genes with predicted functions that could potentially enhance cell survival and growth, including homologs of *aadE* (which confers aminoglycoside resistance [43]), a LexA repressor (involved in the SOS response regulation [44]) and 23S rRNA methyltransferase RlmN (that could impact on cellular growth [45]). It has predicted transposases and conjugation transfer genes as well as homologs of plasmid maintenance and replication protein encoding genes; *parA* and *parB*, and *repA*, suggesting this MGE is also a composite with several origins as determined for other *C. difficile* transposons, Tn*9194* and Tn*6103* [34, 42]. Interestingly, the amino acid sequence of AadE was 100% identical to that of plasmid-carried *aadE* genes in *Campylobacter jejuni* (YP_009079621) and *Pediococcus acidilactic* (YP_001965484), and is present in several Firmicutes *sp*. sequences from WGS projects, further supporting prior observations that this resistance can transmit between bacterial genera [46]. To determine the carriage of Tn*6293* in *C. difficile*, its sequence was searched using BLASTn against *C. difficile* (taxid 1496) sequences. Homologous regions were found in the genomes of three of the seven isolates that are related to M120 (Additional file 2: Table S2); E1 and T5 (R126, human isolates) and NAP08 (R078, human isolate) [21]. To determine its potential origin, the nt sequence was searched against the NCBI nt/nr db. It has similarity to regions in *Eubacterium* and *Ruminococcus* *spp.* genomes. The shared nt sequence similarity is primarily located in genes whose predicted products are involved in genetic element mobilisation and maintenance functions. These include a serine recombinase (CD105HS27_00591), DNA binding and mobilization proteins (CD105HS27_00611 and CD105HS27_00612) and plasmid recombinase (CD105HS27_00634). Both *Eubacterium sp.* and Ruminococcus *sp. * belong to the same order as *C. difficile*, the *Clostridiales*, and the shared sequence similarity observed supports previous findings of MGEs being exchanged between these genera [25].

Both genomes carry a predicted R-type bacteriocin. R-type bacteriocins resemble phage tail-like particles (PTLPs) and have genes predicted to encode proteins involved in structural roles for tail assembly. However, they lack predicted capsid genes and thus are not a complete virion particle. These bacteriocins, or PTLPs, have been observed in culture supernatants of diverse isolates [9, 47, 48], and been used either as typing tools or to determine their use as alternative therapeutics [49, 50]. Due to the specificity required of proteins that target the cell surface, obtaining sequence information from the genomes of clinically relevant strains could aid in using a synthetic biology approach for designer antimicrobials; this has been demonstrated for the bacteriocin carried in a R027 isolate [51], with subsequent genetic modification for enhancing its antimicrobial application [52].

It is not possible to conclude whether these strains have transferred from the environment to the patients or vice versa from the comparisons we have performed here based on a sample size of two. However, the putative origins of these CTns have been examined based on sequence homology. Tn*6164* and Tn*6293* are clearly distinct from one another, and to known elements in other bacterial species. For example, for Tn*6164*, similarity to other sequences is split over the length of the transposon into at least two major regions: the phage containing region is most closely related to a single *Clostridium difficile* genome Z31 (CP013196.1) based on a nt identity of 93% covering 35% of its length. In the same region, the next most closely related elements are found in the complete genome of *Thermoanaerobacter *  *spp.* (CP002210 and CP000923.1) and a draft genome of *Clostridium bornimense* (GCA_000577895). *Thermoanaeroacter* strains were originally isolated from anaerobic enrichments with environmental samples from subsurface. *C. bornimense* is a hydrogen producing *Clostridium* and this species does not have an associated history with human infections, but isolated from a laboratory bioreactor [53]. The second region of the transposon has homology to *Streptococcus* and *Anaerococcus* *spp*. In contrast, Tn*6293* showcases sequence similarity in multiple regions across its full length to different bacterial genera including *Ruminococcus*, *Clostridium* and *Eubacterium* *spp*. It is interesting that the second region of homology in Tn*6164* is to pathogenic species. However, as this is based on few sequences, it is not possible to conclusively state this has been acquired while in clinics despite its absence from CD105HS27 (and thus infer CD105HS27 has evolved outside of clinics). Whether the two isolates have evolved in isolation is one possibility. SNP analysis has been used to track the transfer of strains across the world [54] and in different reservoirs [19, 54], with estimated mutation rates of 1–2 sites per year, suggesting that the number of substitutions (*n* = 85) we observed here suggests that these two isolates have evolved from one another over some time. Increasing numbers of R078 genomes will aid in determining the movement of strains from clinics to the environment and vice versa, in addition to how these strains further evolve when in different reservoirs.

### Methylome of R078 isolates

To establish genome-wide methylation patterns of the two isolates, the profiles for methylation modifications N4-methylcytosine (^m4^C) and N6-methyladenine (^m6^A) were analysed from the SMRT data [55]. Methylation (the addition of methyl groups to bases) in bacteria may play a regulatory role in terms of gene expression [56], but is also one way that DNA elements can exploit to protect against their degradation by restriction modification systems in the host cell [57]. Both strains M120 and CD105HS27 show adenine methylation of the consensus sequence CAAAAA with high efficiency of target methylation (7484/7579, or 98.75% sites in M120 and 7469/7559 or 98.8% in CD105HS27). This target specificity had been previously assigned to the N6-adenine methyltransferase named M.Cdi25 or Cdi630V (locus tag CD630_27580, protein Id YP_001089271.1) of strain CD630 [22] and is reported in the REBASE database [58]. The respective methyltransferases of M120 (CDM120_RS14295, WP_003422891.1) and CD105HS27 (CD105HS27_02520) are identical and show a 98% identity (565/577) to the CD630 orthologue. Strain M120 showed signatures for a N4 modified cysteine ACGGC methylation target (398/414) and a consensus sequence CGGCNTGTGNNNNNNT was identified but with unknown modified base calls (12/13). In REBASE, the ACGGC target is assigned to two tandem methyltransferases of Tn*6164*, M1.CdiMORFAP (CDM120_RS02255, WP_041160334.1) and M2.CdiMORFAP (CDM120_RS02260, WP_041160335.1). No further modified base was detected in strain CD105HS27. The finding that methylation pattern of ^m4^C GCCGT/ACGGC was absent in CD105HS27 may be explained by the absence of Tn*6164* and both these two methyltransferases. In contrast, both M120 and CD105HS27 encode CdiMORFEP, a homolog of M.CdiG46II (amino acid identity of 565/577 (98%)) which is predicted to recognise CAAAAA sites. Three further predicted methylases on Tn*6164* are present in M120 [34] and absent from CD105HS27, as the latter lacks this mobile element. While it was expected for the two Tn*6164* ^m5^C methyltransferases M.CdiMORFBP (CDM120_RS02360, WP_041160353.1) and M.CdiMORFCP (CDM120_RS02725, WP_041160386.1) to show no signature on the SMRT dataset, we would have expected to identify a signature for the putative ^m6^A methyltransferase (CDM120_RS02520, WP_000662263.1). The fact that no additional adenine methylation pattern was detected could be due to one of many reasons including target identity of this enzyme and M.Cdi25/Cdi630V, lack of expression of the enzyme in CD105HS27 or inappropriate annotation of predicted CDSs.

Just as there are different sets of methylation genes functional in *C. difficile*, strains carry genes encoding multiple restriction enzymes [59]. It is of interest to note that despite the fact that M120 and CD105HS27 are highly related, they share only core genome methylation systems as the adenine methylase above or the McrBC system, as they do with the strain CD630. This is due to the fact that the majority of methyltransferases are in Tn*6164* which is absent from CD105HS27. In addition to methylation Restriction Modification (RM) systems, MGEs have other defence systems against super-infection [60]. Here, Tn*6164* carries three putative methylase genes on the transposon region and two on the prophage region of the element. The two sequenced strains were also found to contain defence mechanisms to combat RM systems, notably, Tn*6190* carries *ardA* which encodes ArdA, an anti-restriction protein for type I restriction systems [61]. Whether this system is active remains to be determined, but evidently there are multiple mechanisms employed by MGE in *C. difficile* to be maintained.

### CRISPR/Cas system of M120 and CD105HS27

Immunity to phage infection can also be conferred via the CRISPR (Clusters of Regularly Interspaced Palindromic Repeats)/Cas system which works as an RNA based interference against invading DNA elements [62], but also may act as regulatory machinery for other aspects of the cell biology and genome evolution [63]. The function of the CRISPR/Cas system depends on the action of CRISPR associated (Cas) proteins that are highly diverse in operons across prokaryotes, and ultimately involves the processing and matching of spacers to target DNA with its subsequent restriction [64]. It comprises of arrays that have conserved direct repeat (DR) sequences that flank spacer sequences. Spacers are homologous to phage or plasmid sequences as have been incorporated into arrays following unsuccessful past invasions, and in this way they can provide information about past interactions with such elements [65].

In this study, six CRISPR arrays and three cassettes of Cas genes were identified in each genome. Two Cas gene operons belonged to the I-B/TNeap group and contained all gene components to be functionally complete [64], and the third set comprised of *cas6*, *cas7*, *cas5* and *cas3*, but lacked *cas1* and *cas2*. Multiple *cas* sets within a single genome, of both complete and incomplete operons, have been described previously in *C. difficile* strains CD630 [66] and R20291, but it appears unusual that these two isolates have two complete yet distinct cassettes. The two complete sets are adjacent to CRISPR arrays CRISPR 4 and CRISPR 5.

**Fig. 3:**
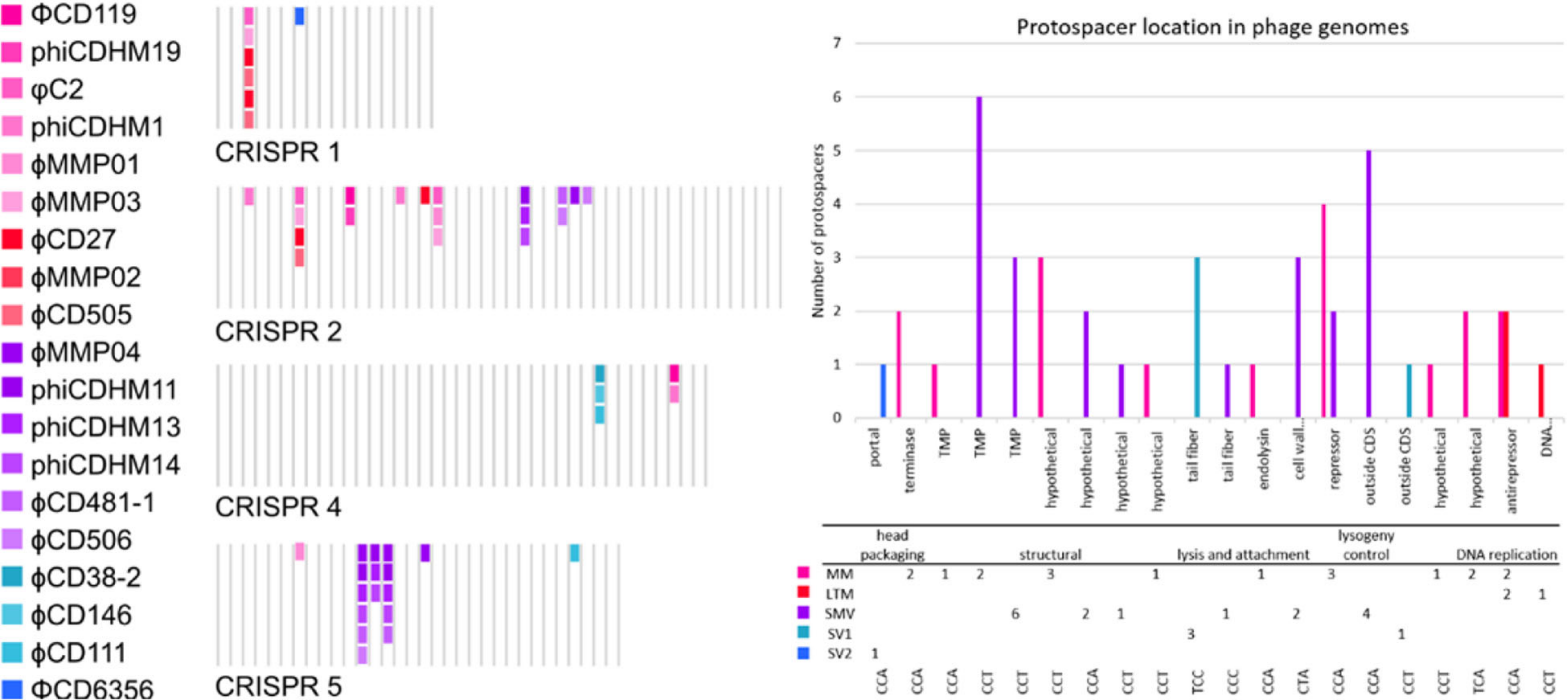
CRISPR spacer content with perfect matches to *C. difficile* phages. *Left*. Positions of spacers for each array with matches to the 18 phages (key coloured according to groups of medium myoviruses (MMs), long tailed myoviruses (LTMs), small myoviruses (SMVs) and siphoviruses (SVs)). The arrays show clear differences in terms of protospacer content with spacers that match to multiple phages. *Right*. Histogram showing the matches to protospacers in phage genes encoding portal, terminase, tape measure (TMP), tail fiber, cell wall hydrolase, repressor, anti-repressor, DNA binding and hypothetical proteins in addition to those outside predicted CDSs with their respective frequencies, and the table below corresponds to the gene’s functional region in the phage genome, phage type and the consensus Protospacer Adjacent Motifs (PAMs) detected

**Fig. 4:**
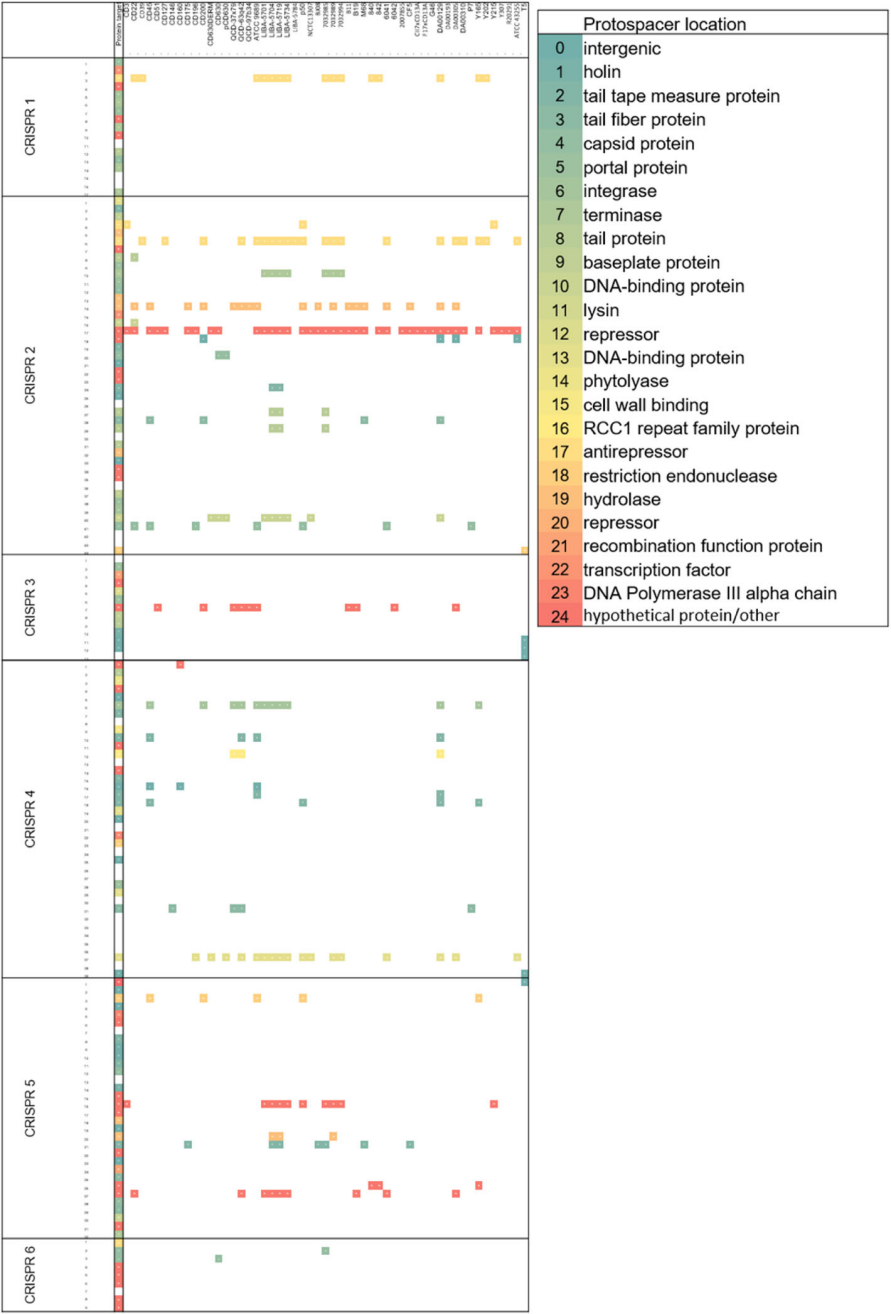
CRISPR spacer content with perfect matches to *C. difficile* isolate genomes. The spacer sequences from the 6 CRISPR arrays (on y axis). Protospacer locations (x axis) are shown in first column from perfect and imperfect matches for annotation (details in figure key). The next 53 columns contain perfect matches between spacers and corresponding *C. difficile* bacterial isolate sequences, coloured according to protospacer location (see key). The protospacer locations include those in conserved prophage genes. A total of 201 perfect matches were identified, with the spacer with most protospacers (*n* = 39) identified for CRISPR_2_17, in a phage protein of unknown function

The six CRISPR arrays are conserved between the two isolates. Five of the arrays have identical spacer contents with 17 (CRISPR_1), 44 (CRISPR 2), 13 (CRISPR 3), 32 (CRISPR 5) and 9 (CRISPR 6) spacers. The remaning array, CRISPR 4, has one additional spacer in CD105HS27 than M120, with 39 and 38 spacers, respectively (spacer number 12, indicated in by Additional file 3: Table S5. by asterisk). Previously, we showed that spacers targeted *C. difficile* phages [66]. Here, we searched spacers from the six arrays against 20 *C. difficile* phage genomes (Fig. 3, Additional file 4: Table S3). Of the total 154 spacers present in both isolates, 19 spacers have at least one identical match to a phage sequence from 18 phages. Perfect matches were identified between spacers and phage sequences from all arrays, except CRISPR arrays 3 and 6. Spacers with matches were located throughout the arrays, but differed with regards to location and type of phage (Fig. 3). We focused on perfect matches as phages phiCDHM1, phiCDHM19, phiCDHM14 and phiCDHM13 do not produce lysis of either strain [22]. To identify matches for the remaining spacers and to a wider range of DNA sequences, we searched the viral and plasmid databases in CRISPRTarget [67], the metaviromic datasets publically available on MetaVir [68] and *C. difficile* genomes (Additional file 4: Table S3 and Additional file 5: Table S4). We did not detect any perfect matches to the viromic datasets, but identified matches for spacers from all six CRISPR arrays to prophage and phage-like genes in the *C. difficile* bacterial genomes (Fig. 4, Additional file 3: Table S5). It has been found that CRISPR systems may also have regulatory roles in genomes [69]. To identify if there were spacers that matched to genomic sequence, we searched the genome of CD105HS27 and identified one perfect match for a spacer in CRISPR 6. The protospacer sequence is located in CD105HS27_02420, a gene encoding a putative carboxylase. This does not have either of the previously identified CCT or CCA Protospacer Adjacent Motif (PAM) sequences [66] so whether this has a functional role is unknown.

We see that in *C. difficile*, CRISPR arrays appear to undergo horizontal exchange between strains via their presence on MGEs, including prophage, plasmids and the *C. difficile sigK* intervening (skin^Cd^) element [18, 66]. In the genome of *C. cellulolyticum* H10, two CRISPR arrays are proximal to a transposase gene which suggests that recombination events could shift immunity profiles via the introduction of novel arrays with new spacer content [70]. Similarly in M120 and CD105S27, two of the arrays, CRISPR 1 and CRISPR 2, are in proximity to CDSs that suggest past integration events containing either integrase or transposase domains. Whether these genes still function and these regions are mobile is not clear from annotation alone. However, these findings of arrays on MGE and signatures of past integration events nearby suggest that arrays could move following genome insertion and excision events by a variety of mechanisms.

## Conclusions

SMRT technology has been used to generate near complete genomes for two R078 strains, allowing the comparison of clinical and environmental isolates. The two genomes differ in chromosomal structure and number of ribosomal operons. Additionally, the two genomes differ in the carriage of two transposons, Tn*6164* in M120 and Tn*6293* in CD105H27, which we suggest are termed as putative conjugative transposons CTn*6164* and CTn*6293*.

The majority of unique genes are carried on the two putative CTns and include predicted methylases. The methylome analysis for each genome suggests a vastly different methylation pattern with no consensus ^m4^C motif in CD105HS27 detected. This likely impacts the immunity of each isolate to DNA elements including phages, and to the type of HGT that may occur for each. In contrast, their CRISPR/Cas systems are highly similar with only one spacer different between the two. Our findings support previous work that the CRISPR/Cas and RM systems are not mutually exclusive [71], and show this indeed appears to be the case in *C. difficile*.

## Methods

### Bacterial genomic DNA extraction

Bacterial genomic DNA (gDNA) extraction was performed using 1 ml overnight culture from a single colony grown in Brain Heart Infusion (BHI) broth (Oxoid, UK). DNA was extracted using a Qiagen GenomicTip 500/G kit (Qiagen, UK) following the manufacturer’s instructions. Pulsed Field Gel Electrophoresis was performed to assess gDNA degradation, with 100 ul of each sample separated on a 1% Agarose gel (Manufacturer info) for 18 h at 6 V. Gels were stained with 10 ul of ethidium bromide and visualised using UV G Box, Syngene. Sample gDNA quantity and quality was measured using by Qubit assay on a Qubit fluorometer (Life Technologies, USA) according to the manufacturer’s instructions, and by measuring absorbance at 260 nm and 280 nm using a Nanodrop Spectrophotometer (Thermo Scientific, UK).

### Genome sequencing and bioinformatics analysis

Genomic DNA sequencing using a SMRT Pacific Biosciences platform was performed at the Centre for Genomic Research, University of Liverpool. SMRTbell libraries were prepared by Margaret A. Hughes with 3 SMRT cells used per library for sequencing. High quality genome assemblies were generated using HGAP (Hierarchal Genome Assembly Processer) as part of the SMRT Portal and methylation patterns detected. Contig structure and plasmid identification was performed from dotplots generated using Gepard [72].

Genomes were visualised using Artemis Genome Browser [73]. Coverage was determined from alignment of the corrected reads to the final assembly using BWA-SW [74], and samtools for index and conversion of file formats [75]. Coverage was assessed using Qualimap v.1.0 [76] and coverage plots were generated using the Artemis DNAplotter perl script [77]. Genome annotation was performed using PROKKA v1.7 [78], with a custom guide database containing proteins from the reference genome of M120 (accession NC_017174.1). RNA genes were predicted using RNAmmer v1.2 [79]. *In silico* ribotypes profiles were predicted using the oligonucleotide sequences from Bidet et al. [80]. Shared gene content was identified with blast + v2.2.28 using blast-all-v-all [81]. This publication made use of the *Clostridium difficile* Multi Locus Sequence Typing website (http://pubmlst.org/cdifficile/) developed by Keith Jolley and sited at the University of Oxford [82]. The characterised *C. difficile* CD630 CTns were used as a reference set for the identified of similar MGEs by BLASTn. Whole genome alignment and single nucleotide differences were generated using MAUVE v.2.4.0 [83]. Average nucleotide identity was calculated following the method described in [38], using the online web based tool which can be accessed at http://enve-omics.ce.gatech.edu/ani/ with parameters of min. length 700 bp, min. identity 70% and min. alignment 50. Dotplot analysis was generated using Gepard [72]. Genome comparison maps were generated using EasyFig v.2.2.2 [84]. Restriction modification systems were analysed using entries from REBASE (the Restriction Enzyme database) [58]. Prophage regions were predicted using PHAST [85]. CRISPR arrays were identified using CRISPRfinder [86], and the genomes CRISPR content compared using CRISPRcompar [87]. Spacer sequences were searched against the GenBank-Phage, RefSeq-Plasmid, RefSeq-Viral and Genbank-Environmental databases (accessed 1/10/2015) using CRISPRTarget [67] in addition to virus metagenome datasets (Additional file 5: Table S4). Spacer protein targets were identified using a curated approach based on annotations on the NCBI genome browser at locations identified from the CRISPRTarget search. Where no annotation was available from perfect spacer-target matches on CRISPRTarget, consensus annotations from imperfect matches (up to 7 mismatches) were used.

## Additional files

Additional file 1: Table S1.
*In silico* ribotype profiles for the *C. difficile* genomes. (DOCX 12 kb)
Additional file 2: Table S2. Novel transposon sequence similarity in *C. difficile* strains. (DOCX 12 kb)
Additional file 3: Table S5. CRISPR spacer matches to *C. difficile* genomic sequences. (DOCX 27 kb)
Additional file 4: Table S3. Genome sequences used in this study. (DOCX 13 kb)
Additional file 5: Table S4. Viral metagenome datasets used for protospacer identification. (DOCX 13 kb)

## Abbreviations

Bp: Basepairs
Cas: CRISPR associated proteins
CDI: *Clostridium difficile* infection
CDS: Coding DNA sequence
CDT: *Clostridium difficile* binary toxin
CRISPR: Clustered regularly interspaced short palindromic repeats
CTn: Conjugative transposon
DNA: Deoxyribonucleic acid
DR: Direct repeat
GC: Guanine cytosine
gDNA: Genomic DNA
Kbp: Kilobasepairs
LTM: Long tailed myovirus
^m4^C: N4-methylcytosine
^m5^C: N5-methylcytosine
^m6^A: N6-methyladenine
MGE: Mobile genetic element
MLST: MultiLocus sequence typing
MM: Medium myovirus
NCBI nr/nt db: National Center for Biotechnology Information non-redundant nucleotide database
Nm: Nanometres
Nt: Nucleotide
PaLoc: Pathogenicity locus
PAM: Protospacer adjacent motif
PCR: Polymerase chain reaction
REBASE: The Restriction Enzyme database
RM: Restriction modification
RNA: Ribonucleic acid
rRNA: Ribosomal ribonucleic acid
skin^cd^: *Clostridium difficile sigK* intervening element
SMRT: Single molecule real time
SMV: Small myovirus
SNP: Single nucleotide polymorphism
SV: Siphovirus
Tn: Transposon
tRNA: Transfer ribonucleic acid
UV: Ultraviolet

## References

[1] Lawson PA, Citron DM, Tyrrell KL, Finegold SM (2016). Reclassification of Clostridium difficile as Clostridioides difficile (Hall and O’Toole 1935) Prévot 1938. Anaerobe.

[2] Leffler DA, Lamont JT (2015). Clostridium difficile Infection. New Engl J Med.

[3] Gerding DN (2004). Clindamycin, cephalosporins, fluoroquinolones, and Clostridium difficile-associated diarrhea: This is an antimicrobial resistance problem. Clin Infect Dis.

[4] Eyre DW, Griffiths D, Vaughan A, Golubchik T, Acharya M, O’Connor L, Crook DW, Walker AS, Peto TE (2013). Asymptomatic Clostridium difficile colonisation and onward transmission. PLoS One.

[5] Metcalf DS, Costa MC, Dew WMV, Weese JS (2010). Clostridium difficile in vegetables, Canada. Lett Appl Microbiol.

[6] Weese JS, Avery BP, Rousseau J, Reid-Smith RJ (2009). Detection and Enumeration of Clostridium difficile Spores in Retail Beef and Pork. Appl Environ Microbiol.

[7] Al Saif N, Brazier JS (1996). The distribution of Clostridium difficile in the environment of South Wales. J Med Microbiol.

[8] Zidaric V, Beigot S, Lapajne S, Rupnik M (2010). The occurrence and high diversity of Clostridium difficile genotypes in rivers. Anaerobe.

[9] Hargreaves KR, Colvin HV, Patel KV, Clokie JJP, Clokie MRJ (2013). Genetically Diverse Clostridium difficile Strains Harboring Abundant Prophages in an Estuarine Environment. Appl Environ Microbiol.

[10] Del Mar Gamboa M, Rodriguez E, Vargas P (2005). Diversity of mesophilic clostridia in Costa Rican soils. Anaerobe.

[11] Pasquale V, Romano VJ, Rupnik M, Dumontet S, Ciznar I, Aliberti F, Mauri F, Saggiomo V, Krovacek K (2011). Isolation and characterization of Clostridium difficile from shellfish and marine environments. Folia Microbiol.

[12] Metcalf D, Avery BP, Janecko N, Matic N, Reid-Smith R, Weese JS (2011). Clostridium difficile in seafood and fish. Anaerobe.

[13] Miller MA, Byrne BA, Jang SS, Dodd EM, Dorfmeier E, Harris MD, Ames J, Paradies D, Worcester K, Jessup DA (2010). Enteric bacterial pathogen detection in southern sea otters (Enhydra lutris nereis) is associated with coastal urbanization and freshwater runoff. Vet Res.

[14] Goorhuis A, Bakker D, Corver J, Debast S, Harmanus C, Notermans D, Bergwerff A, Dekker F, Kuijper E (2008). Emergence of Clostridium difficile infection due to a new hypervirulent strain, polymerase chain reaction ribotype 078. Clin Infect Dis.

[15] Smits WK (2013). Hype or hypervirulence. Virulence.

[16] Barbut F, Rupnik M (2012). 027, 078, and Others: Going Beyond the Numbers (and Away From the Hypervirulence). Clin Infect Dis.

[17] Cairns M, Stabler R, Shetty N, Wren B (2012). The continually evolving Clostridium difficile species. Future Microbiol.

[18] Sebaihia M, Wren B, Mullany P, Fairweather N, Minton N, Stabler R, Thomson N, Roberts A, Cerdeno-Tarrraga A, Wang H (2006). The multidrug-resistant human pathogen Clostridium difficile has a highly mobile, mosaic genome. Nat Genet.

[19] Knetsch CW, Connor TR, Mutreja A, Van Dorp SM, Sanders IM, Browne HP, Harris D, Lipman L, Keessen EC, Corver J (2014). Whole genome sequencing reveals potential spread of Clostridium difficile between humans and farm animals in the Netherlands, 2002 to 2011. Eurosurveillance.

[20] He M, Sebaihia M, Lawley T, Stabler R, Dawson L, Martin M, Holt K, Seth-Smith H, Quail M, Rance R (2010). Evolutionary dynamics of Clostridium difficile over short and long time scales. Proc Natl Acad Sci U S A.

[21] Kurka H, Ehrenreich A, Ludwig W, Monot M, Rupnik M, Barbut F, Indra A, Dupuy B, Liebl W (2014). Sequence similarity of Clostridium difficile strains by analysis of conserved genes and genome content is reflected by their ribotype affiliation. PLoS One.

[22] Hargreaves KR, Otieno JR, Thanki A, Blades MJ, Millard AD, Browne HP, Lawley TD, Clokie MRJ (2015). As clear as mud? Determining the diverity and prevalance of prophages in the draft genomes of estuarine isolates of Clostridium difficile. Genome Biol Evol.

[23] Knight DR, Elliott B, Chang BJ, Perkins TT, Riley TV (2015). Diversity and Evolution in the Genome of Clostridium difficile. Clin Microbiol Rev.

[24] Amy J, Johanesen P, Lyras D (2015). Extrachromosomal and integrated genetic elements in Clostridium difficile. Plasmid.

[25] Mullany P, Allan E, Roberts AP (2015). Mobile genetic elements in Clostridium difficile and their role in genome function. Res Microbiol.

[26] Vedantam G, Clark A, Chu M, McQuade R, Mallozzi M, Viswanathan VK (2012). Clostridium difficile infection: toxins and non-toxin virulence factors, and their contributions to disease establishment and host response. Gut Microbes.

[27] Luo Y, Huang C, Ye J, Fang W, Gu W, Chen Z, Li H, Wang X, Jin D (2016). Genome Sequence and Analysis of Peptoclostridium difficile Strain ZJCDC-S82. Evol Bioinforma.

[28] Van Eijk E, Anvar SY, Browne HP, Leung WY, Frank J, Schmitz AM, Roberts AP, Smits WK (2015). Complete genome sequence of the Clostridium difficile laboratory strain 630Deltaerm reveals differences from strain 630, including translocation of the mobile element CTn5. BMC Genomics.

[29] Gaulton T, Misra R, Rose G, Baybayan P, Hall R, Freeman J, Turton J, Picton S, Korlach J, Gharbia S (2015). Complete Genome Sequence of the Hypervirulent Bacterium Clostridium difficile Strain G46, Ribotype 027. Genome Announc.

[30] Riedel T, Bunk B, Wittmann J, Thurmer A, Sproer C, Gronow S, Liesegang H, Daniel R, Overmann J (2015). Complete Genome Sequence of the Clostridium difficile Type Strain DSM 1296T. Genome Announc.

[31] Riedel T, Bunk B, Thurmer A, Sproer C, Brzuszkiewicz E, Abt B, Gronow S, Liesegang H, Daniel R, Overmann J (2015). Genome Resequencing of the Virulent and Multidrug-Resistant Reference Strain Clostridium difficile 630. Genome Announc.

[32] Gyorfy Z, Draskovits G, Vernyik V, Blattner FF, Gaal T, Posfai G (2015). Engineered ribosomal RNA operon copy-number variants of E-coli reveal the evolutionary trade-offs shaping rRNA operon number. Nucleic Acids Res.

[33] Janezic S, Ocepek M, Zidaric V, Rupnik M (2012). Clostridium difficile genotypes other than ribotype 078 that are prevalent among human, animal and environmental isolates. BMC Microbiol.

[34] Corver J, Bakker D, Brouwer MS, Harmanus C, Hensgens MP, Roberts AP, Lipman LJ, Kuijper EJ, Van Leeuwen HC (2012). Analysis of a Clostridium difficile PCR ribotype 078 100 kilobase island reveals the presence of a novel transposon, Tn6164. BMC Microbiol.

[35] Santagati M, Iannelli F, Cascone C, Campanile F, Oggioni MR, Stefani S, Pozzi G (2003). The novel conjugative transposon tn1207.3 carries the macrolide efflux gene mef (A) in Streptococcus pyogenes. Microb Drug Resist.

[36] Iannelli F, Santagati M, Santoro F, Oggioni MR, Stefani S, Pozzi G (2014). Nucleotide sequence of conjugative prophage Φ1207.3 (formerly Tn1207.3) carrying the mef (A)/msr (D) genes for efflux resistance to macrolides in Streptococcus pyogenes. Front Microbiol.

[37] Johnson SR, Romig WR (1981). Vibrio cholerae conjugative plasmid pSJ15 contains transposable prophage dVcA1. J Bacteriol.

[38] Goris J, Konstantinidis KT, Klappenbach JA, Coenye T, Vandamme P, Tiedje JM (2007). DNA-DNA hybridization values and their relationship to whole-genome sequence similarities. Int J Syst Evol Microbiol.

[39] Feiner R, Argov T, Rabinovich L, Sigal N, Borovok I, Herskovits AA (2015). A new perspective on lysogeny: prophages as active regulatory switches of bacteria. Nat Rev Microbiol.

[40] Griffiths D, Fawley W, Kachrimanidou M, Bowden R, Crook DW, Fung R, Golubchik T, Harding RM, Jeffery KJM, Jolley KA (2010). Multilocus Sequence Typing of Clostridium difficile. J Clin Microbiol.

[41] Stabler RA, Dawson LF, Valiente E, Cairns MD, Martin MJ, Donahue EH, Riley TV, Songer JG, Kuijper EJ, Dingle KE (2012). Macro and micro diversity of Clostridium difficile isolates from diverse sources and geographical locations. PLoS One.

[42] Brouwer MS, Warburton PJ, Roberts AP, Mullany P, Allan E (2011). Genetic organisation, mobility and predicted functions of genes on integrated, mobile genetic elements in sequenced strains of Clostridium difficile. PLoS One.

[43] Shaw KJ, Rather PN, Hare RS, Miller GH (1993). Moleclar genetics of aminoglycoside resistance genes and familial relationships of the aminoglycoside-modifying enzymes. Microbiol Rev.

[44] Butala M, Zgur-Bertok D, Busby SJW (2009). The bacterial LexA transcriptional repressor. Cell Mol Life Sci.

[45] Toh S-M, Xiong L, Bae T, Mankin AS (2008). The methyltransferase YfgB/RlmN is responsible for modification of adenosine 2503 in 23S rRNA. RNA.

[46] Courvalin P (1994). Transfer of antibiotic-resistance genes between Gram-positive and Gram-negative bacteria. Antimicrob Agents Chemother.

[47] Nale J, Shan J, Hickenbotham P, Fawley W, Wilcox M, Clokie M (2012). Diverse Temperate Bacteriophage Carriage in Clostridium difficile 027 Strains. PLoS One.

[48] Fortier L, Moineau S (2007). Morphological and genetic diversity of temperate phages in Clostridium difficile. Appl Environ Microbiol.

[49] Sell TL, Schaberg DR, Fekety FR (1983). Bacteriophage and bacteriocin typing scheme for Clostridium difficile. J Clin Microbiol.

[50] Sangster W, Hegarty JP, Stewart DB (2015). Phage tail-like particles kill Clostridium difficile and represent an alternative to conventional antibiotics. Surgery.

[51] Gebhart D, Williams SR, Bishop-Lilly KA, Govoni GR, Willner KM, Butani A, Sozhamannan S, Martin D, Fortier L-C, Scholl D (2012). Novel High-Molecular-Weight, R-Type Bacteriocins of Clostridium difficile. J Bacteriol.

[52] Gebhart D, Lok S, Clare S, Tomas M, Stares M, Scholl D, Donskey CJ, Lawley TD, Govoni GR (2015). A modified R-type bacteriocin specifically targeting Clostridium difficile prevents colonization of mice without affecting gut microbiota diversity. MBio.

[53] Hahnke S, Striesow J, Elvert M, Mollar XP, Klocke M (2014). Clostridium bornimense sp. nov., isolated from a mesophilic, two-phase, laboratory-scale biogas reactor. Int J Syst Evol Microbiol.

[54] He M, Miyajima F, Roberts P, Ellison L, Pickard D, Martin M, Connor T, Harris S, Fairley D, Bamford K (2013). Emergence and global spread of epidemic healthcare-associated *Clostridium difficile*. Nat Genet.

[55] Flusberg BA, Webster DR, Lee JH, Travers KJ, Olivares EC, Clark TA, Korlach J, Turner SW (2010). Direct detection of DNA methylation during single-molecule, real-time sequencing. Nat Methods.

[56] Marinus MG, Casadesus J (2009). Roles of DNA adenine methylation in host-pathogen interactions: mismatch repair, transcriptional regulation, and more. FEMS Microbiol Rev.

[57] Oliveira PH, Touchon M, Rocha EPC (2014). The interplay of restriction-modification systems with mobile genetic elements and their prokaryotic hosts. Nucleic Acids Res.

[58] Roberts RJ, Vincze T, Posfai J, Macelis D (2015). REBASE-a database for DNA restriction and modification: enzymes, genes and genomes. Nucleic Acids Res.

[59] Stabler RA, He M, Dawson L, Martin M, Valiente E, Corton C, Lawley TD, Sebaihia M, Quail MA, Rose G (2009). Comparative genome and phenotypic analysis of Clostridium difficile 027 strains provides insight into the evolution of a hypervirulent bacterium. Genome Biol.

[60] Kobayashi I (2001). Behavior of restriction-modification systems as selfish mobile elements and their impact on genome evolution. Nucleic Acids Res.

[61] Thomas AT, Brammar WJ, Wilkins BM (2003). Plasmid R16 ArdA protein preferentially targets restriction activity of the type I restriction-modification system EcoKI. J Bacteriol.

[62] Barrangou R, Fremaux C, Deveau H, Richards M, Boyaval P, Moineau S, Romero D, Horvath P (2007). CRISPR provides acquired resistance against viruses in prokaryotes. Science.

[63] Westra ER, Buckling A, Fineran PC (2014). CRISPR-Cas systems: beyond adaptive immunity. Nat Rev Microbiol.

[64] Makarova KS, Wolf YI, Alkhnbashi OS, Costa F, Shah SA, Saunders SJ, Barrangou R, Brouns SJJ, Charpentier E, Haft DH (2015). An updated evolutionary classification of CRISPR-Cas systems. Nat Rev Microbiol.

[65] Marraffini LA, Sontheimer EJ (2010). Self versus non-self discrimination during CRISPR RNA-directed immunity. Nature.

[66] Hargreaves KR, Flores CO, Lawley TD, Clokie MR (2014). Abundant and diverse clustered regularly interspaced short palindromic repeat spacers in Clostridium difficile strains and prophages target multiple phage types within this pathogen. MBio.

[67] Biswas A, Gagnon JN, Brouns SJJ, Fineran PC, Brown CM (2013). CRISPRTarget: Bioinformatic prediction and analysis of crRNA targets. RNA Biol.

[68] Roux S, Faubladier M, Mahul A, Paulhe N, Bernard A, Debroas D, Enault F (2011). Metavir: a web server dedicated to virome analysis. Bioinformatics.

[69] Sampson TR, Weiss DS (2014). CRISPR-Cas systems: new players in gene regulation and bacterial physiology. Front Cell Infect Microbiol.

[70] Brown SD, Nagaraju S, Utturkar S, De Tissera S, Segovia S, Mitchell W, Land ML, Dassanayake A, Koepke M (2014). Comparison of single-molecule sequencing and hybrid approaches for finishing the genome of Clostridium autoethanogenum and analysis of CRISPR systems in industrial relevant Clostridia. Biotechnology for Biofuels.

[71] Dupuis M-E, Villion M, Magadan AH, Moineau S (2013). CRISPR-Cas and restriction-modification systems are compatible and increase phage resistance. Nat Commun.

[72] Krumsiek J, Arnold R, Rattei T (2007). Gepard: a rapid and sensitive tool for creating dotplots on genome scale. Bioinformatics.

[73] Rutherford K, Parkhill J, Crook J, Horsnell T, Rice P, Rajandream M, Barrell B (2000). Artemis: sequence visualization and annotation. Bioinformatics.

[74] Li H, Durbin R (2010). Fast and accurate long-read alignment with Burrows-Wheeler transform. Bioinformatics.

[75] Li H, Durbin R (2009). Fast and accurate short read alignment with Burrows-Wheeler transform. Bioinformatics.

[76] Garcia-Alcalde F, Okonechnikov K, Carbonell J, Cruz LM, Goetz S, Tarazona S, Dopazo J, Meyer TF, Conesa A (2012). Qualimap: evaluating next-generation sequencing alignment data. Bioinformatics.

[77] Carver T, Thomson N, Bleasby A, Berriman M, Parkhill J (2009). DNAPlotter: circular and linear interactive genome visualization. Bioinformatics.

[78] Seamann T (2014). Prokka: rapid prokaryotic genome annotation. Bioinformatics.

[79] Lagesen K, Hallin P, Rodland EA, Staerfeldt H-H, Rognes T, Ussery DW (2007). RNAmmer: consistent and rapid annotation of ribosomal RNA genes. Nucleic Acids Res.

[80] Bidet P, Barbut F, Lalande V, Burghoffer B, Petit JC (1999). Development of a new PCR-ribotyping method for Clostridium difficile based on ribosomal RNA gene sequencing. FEMS Microbiol Lett.

[81] Camacho C, Coulouris G, Avagyan V, Ma N, Papadopoulos J, Bealer K, Madden TL (2009). BLAST plus: architecture and applications. Bmc Bioinformatics.

[82] Jolley KA, Maiden MCJ (2010). BIGSdb: Scalable analysis of bacterial genome variation at the population level. BMC Bioinformatics.

[83] Darling A, Mau B, Blattner F, Perna N (2004). Mauve: Multiple alignment of conserved genomic sequence with rearrangements. Genome Res.

[84] Alikhan N-F, Petty NK, Ben Zakour NL, Beatson SA (2011). BLAST Ring Image Generator (BRIG): simple prokaryote genome comparisons. BMC Genomics.

[85] Zhou Y, Liang Y, Lynch KH, Dennis JJ, Wishart DS (2011). PHAST: A Fast Phage Search Tool. Nucleic Acids Res.

[86] Grissa I, Vergnaud G, Pourcel C (2007). CRISPRFinder: a web tool to identify clustered regularly interspaced short palindromic repeats. Nucleic Acids Res.

[87] Grissa I, Vergnaud G, Pourcel C (2008). CRISPRcompar: a website to compare clustered regularly interspaced short palindromic repeats. Nucleic Acids Res.

