# Supplementary material for: Use of single molecule sequencing for comparative genomics of an environmental and a clinical isolate of Clostridium difficile ribotype 078
Source: BMC Genomics. 2016 Dec 13;17:1020. doi: 10.1186/s12864-016-3346-2 (PMC5154133; doi:10.1186/s12864-016-3346-2)
Supplement: Additional file 1: Table S1. — In silico ribotype profiles for the C. difficile genomes. (DOCX 12 kb) [file 12864_2016_3346_MOESM1_ESM.docx]

| **M120 Reference** | **M120** | **CD105HS27** |
| --- | --- | --- |
| 345 | 347 | 347 |
| 346 | 365 | 365 |
| 347 | 367 | 367 |
| 367 | 409 | 367 |
| 409 | 409 | 407 |
| 409 | 451 | 409 |
| 451 | 485 | 409 |
| 485 | 485 | 409 |
| 485 | 485 | 409 |
| 526 | 522 | 451 |
| 527 | 527 | 485 |
|  | 527 | 485 |
|  |  | 485 |
|  |  | 485 |
|  |  | 527 |
|  |  | 527 |
