# Supplementary material for: Use of single molecule sequencing for comparative genomics of an environmental and a clinical isolate of Clostridium difficile ribotype 078
Source: BMC Genomics. 2016 Dec 13;17:1020. doi: 10.1186/s12864-016-3346-2 (PMC5154133; doi:10.1186/s12864-016-3346-2)
Supplement: Additional file 2: Table S2. — Novel transposon sequence similarity in C. difficile strains. (DOCX 12 kb) [file 12864_2016_3346_MOESM2_ESM.docx]

| **Organism** | **Accession** | **Query coverage** | **E value** | | **Identity** |
| --- | --- | --- | --- | --- | --- |
| *C. difficile* T5 genomic scaffold | NZ_HF677387.1 | 47% | | 0 | 100% |
|  | NZ_HF677386.1 | 43% | | 0 | 99% |
| *C. difficile* E1 genomic scaffold | NZ_HF927576.1 | 95% | | 0 | 100% |
| *C. difficile* NAP08 genomic scaffold | NZ_GG770710.1 | 95% | | 0 | 99% |
| *C. clostridioform* 90A6 genomic scaffold | NZ_KB851028.1 | 81% | | 0 | 99% |
