# Supplementary material for: Use of single molecule sequencing for comparative genomics of an environmental and a clinical isolate of Clostridium difficile ribotype 078
Source: BMC Genomics. 2016 Dec 13;17:1020. doi: 10.1186/s12864-016-3346-2 (PMC5154133; doi:10.1186/s12864-016-3346-2)
Supplement: Additional file 3: Table S5. — CRISPR spacer matches to C. difficile genomic sequences. (DOCX 27 kb) [file 12864_2016_3346_MOESM3_ESM.docx]

| **CRISPR** | **Protospacer location** | **Accession for annotation** | ***C. difficile* Genome matches** | **PAM** |
| --- | --- | --- | --- | --- |
|  |  |  |  |  |
| CRISPR 1 | |  |  |  |
| 1 | SPP1 family phage portal protein | AKP41260.1 | - |  |
| 2 | transcriptional regulator, BlaI/MecI/CopY family | CEK40666.1 | - |  |
| 3 | putative antirepressor | YP_529598.1 | CD22 CD39 ATCC 9689 LIBA-5701 LIBA-5704 LIBA-5719 LIBA-5734 7032985 840 842 7032994 7032989 DA00129 Y165 Y202 | 3' TTAACTAT ACCAATGA 5' |
| 4 | conserved hypothetical protein | YP_006990593.1 | - |  |
| 5 | putative phage site-specific integrase | CDS86953.1 | - |  |
| 6 | putative phage terminase, large subunit | CDT82038.1 | - |  |
| 7 | putative phage portal protein | CDL65284.1 | - |  |
| 8 | conserved hypothetical protein | YP_006990605.1 | - |  |
| 9 | putative terminase large subunit protein | CDL65284.1 | - |  |
| 10 | conserved hypothetical protein | YP_004306127.1 | - |  |
| 11 | - | - | - |  |
| 12 | phage major tail , phi13 family protein | WP_021379019.1 | - |  |
| 13 | putative major capsid protein A | YP_004306106.1 | - |  |
| 14 | phage terminase large subunit PBSX family | WP_021373953.1 | - |  |
| 15 | - | - | - |  |
| 16 | - | - | - |  |
| 17 | putative terminase large subunit protein | CDU85288.1 | - |  |
| CRISPR 2 | |  |  |  |
| 1 | DNA-binding protein | CKG88967.1 | - |  |
| 2 | putative tail tape measure protein | CKG88967.1 | - |  |
| 3 | phage baseplate assembly protein | WP_022617936.1 | - |  |
| 4 | putative antirepressor | WP_021359703.1 | CD3 P50 Y215 | 3' ATGTTGGT CTTCACCC 5' |
| 5 | putative LysM | CEK40858.1 | - |  |
| 6 | putative antirepressor | CEK40858.1 | ATCC 43255 Y202 Y165 DA00310 DA00305 DA00129 60417032994 7032989 p50 7032985 LIBA-5784 LIBA-5734 LIBA-5719 LIBA-5704 LIBA-5701 ATCC 9689 QCD-63q42 CD200 CD127 CD39 | 3' GCTATGGT AAGAATAT 5' |
| 7 | putative phage protein | WP_021365855.1 | - |  |
| 8 | psuedo phage tail protein | - | CD22 | 3' GACGAGGT TATATCGA 5' |
| 9 | SPP1 family phage portal protein | AKP41260.1 | - |  |
| 10 | putative terminase large subunit protein | CDW17183.1 | LIBA-5701 LIBA-5704 LIBA-5719 LIBA-5734 7032985 7032989 7032994 | 3' TAGAAGGT CTGAGTTG 5' |
| 11 | SPP1 family phage portal protein | AKP41260.1 | - |  |
| 12 | putative capsid protein | CDW17190.1 | - |  |
| 13 | cell wall hydrolase | WP_009894393.1 | 6503 | 3' GAAAAGGA AAATAGTG 5' |
| 14 | putative endolysin | YP_009032176.1 | CD22 CD45 CD175 CD200 QCD-37x79 QCD-63q42 QCD-97b34 ATCC9689 P50 BJ08 7032989 BI1 BI9 M68 6041 CF5 DA00129 DA00305 | 3' AAAAGGGT ACAGTTGA 5' |
| 15 | putative sigma factor | YP_006990553.1 | - |  |
| 16 | putative phage DNA-binding protein | ACH91363.1 | CD22 | 3' CTTTTGGA AACCAAAG 5' |
| 17 | phage protein | AKP41289.1 | CD3 CD22 6041 6503 2007855 CD45 842 M68 CD51 BI1 BI9 CD127 CD129 CD175 CD196 pCD630 7032989 7032994 ATCC9689 NCTC13307 BJ08 7032985 LIBA-5701 LIBA-5704 LIBA-5719 LIBA-5734 P50 CF5 CII7xCD13A F17xCD13A G46 DA00129 DA00193 DA00305 DA00310 Y165 Y215 Y307 R20291 ATCC43255 | 3' ATTGAGGA AAAAATCA 5' |
| 18 | glycine rich family protein | WP_021389574.1 | CD200 DA00129 DA00305 ATCC43255 | 3' ACTACGGA GAACGAAG 5' |
| 19 | putative tail tape measure protein | WP_021389574.1 | - |  |
| 20 | putative phage capsid protein | YP_659580.1 | pCD630 | 3' TATTTGGA TGAACTCG 5' |
| 21 | intergenic region near integrase | - | - |  |
| 22 | glycine rich family protein | AKP41291.1 | - |  |
| 23 | conserved hypothetical protein | WP_003425642.1 | - |  |
| 24 | putative tail tape measure protein | CDL68830.1 | LIBA-5704 LIBA-5719 | 3' GTTCTGGA CTATTTTA 5' |
| 25 | CRISPR region | - | - |  |
| 26 | intergenic region | - | - |  |
| 27 | phage protein, HK97 gp10 family | CEK40579.1 | LIBA-5704 LIBA-5719 7032985 | 3' TACGAGGT GGGTAAAA 5' |
| 28 | tail fibre protein | YP_006990576.1 | CD45 CD200 M68 DA00129 | 3' GACAAGGA ATAACGCT 5' |
| 29 | Phage protein, HK97 gp10 family | CEK40709.1 | LIBA-5704 LIBA-5719 7032985 | 3' TTTTCGGA ACCTTTTG 5' |
| 30 | - | - | - |  |
| 31 | phage tail protein | WP_032507917.1 | - |  |
| 32 | putative hydrolase | CDS85297.1 | - |  |
| 33 | intergenic region | - | - |  |
| 34 | conserved hypothetical protein | WP_021383732.1 | - |  |
| 35 | pseudo phage tail protein | - | - |  |
| 36 | - | - | - |  |
| 37 | baseplate J-like family protein | CDS85297.1 | - |  |
| 38 | phage portal protein, SPP1 family | AKP41260.1 | - |  |
| 39 | phage integrase | WP_021387472.1 | - |  |
| 40 | phage recombination protein Bet | CKH53715.1 | pCD630 LIBA-5701 LIBA-5704 LIBA-5719 LIBA-5734 NCTC13307 DA00129 | 3' GTTCAGGT TTCCAAAC 5' |
| 41 | phage capsid E family protein | CDIF1296T_00371 | P7 6041 P50 ATCC9689 CD196 CD145 CD22 | 3' CAGTCGTC TTATAGAT 5' |
| 42 | - | - | - |  |
| 43 | - | - | - |  |
| 44 | restriction endonuclease subunit M | WP_009899344.1 | T5 | 3' TACATTTC CGAAATAT 5' |
| CRISPR 3 | |  |  |  |
| 1 | - | - | - |  |
| 2 | SPP1 family phage portal protein | AKP41260.1 | - |  |
| 3 | essential recombination function protein | AKP41233.1 | - |  |
| 4 | pseudo hypothetical gene | NZ_CCEV01000004.1 | - |  |
| 5 | phage repressor | YP_006990554.1 | CD51 CD200 QCD-37x39 QCD-63q42 QCD-97b34 ATCC9689 BI1 BI9 6042 DA00305 | 3' AGAAAGGA GATCTATA 5' |
| 6 | XkdP protein | CDS84765.1 | - |  |
| 7 | conserved hypothetical protein | WP_003425657.1 | - |  |
| 8 | putative baseplate-J protein | CDU85304.1 | - |  |
| 9 | putative phage protein, HK97 gp10 family | CDS87014.1 | - |  |
| 10 | holin | YP_006990580.1 | - |  |
| 11 | intergenic region | - | T5 | 3' TACATTTA CAAAATAT 5' |
| 12 | CRISPR region | - | T5 | 3' TACATTCA CAAAATAT 5' |
| 13 | intergenic region | - | T5 | 3' TATATTTC CAAAATAT 5' |
| CRISPR 4 | |  |  |  |
| 1 | conserved hypothetical protein | WP_021383075.1 | CD160 | 3' AATGTAGT AATCCCTA 5' |
| 2 | phage tail protein | AKP44706.1 | - |  |
| 3 | phytolyase | WP_021425189.1 | - |  |
| 4 | conserved hypothetical protein | WP_021383119.1 | - |  |
| 5 | intergenic region near tail protein | - | - |  |
| 6 | phage integrase | CEK40435.1 | CD45 CD200 QCD-37x79 QCD-63q42 ATCC9689 LIBA-5701 LIBA-5704 LIBA-5719 LIBA-5734 DA00129 Y165 | 3' TTTTTAGT CAACTCTT 5' |
| 7 | putative tape tail measure protein | AKP44699.1 | - |  |
| 8 | - | - | - |  |
| 9 | cell wall binding repeat 2 family protein | WP_021425144.1 | - |  |
| 10 | putative tape tail measure protein | AKP44699.1 | DA00129 ATCC9689 QCD-63q42 CD45 | 3' GATTTAGT TTAAGGTA 5' |
| 11 | hypothetical protein | WP_009894893.1 | QCD-37x79 QCD-63q42 DA00129 | 3' TATAAGTT TATATAGG 5' |
| 12* | RCC1 repeat family protein | WP_021394588.1 | - |  |
| 13 | - | - | - |  |
| 14 | hypothetical protein | WP_042742205.1 | - |  |
| 15 | putative phage tail tape measure protein | AKP44699.1 | - |  |
| 16 | intergenic region near phage tail tape measure protein | - | ATC9689 CD160 CD45 | 3' CACAGAGT CACTAATC 5' |
| 17 | phage tail tape measure protein | AKP44698.1 | ATC9689 DA00129 | 3' AAAGTAGT AAAATTAT 5' |
| 18 | phage tail tape measure protein | WP_021371694.1 | CD45 P50 DA00129 Y165 | 3' TTCTAAGT GTAAAAGA 5' |
| 19 | cold-shock DNA-binding domain-containing protein | WP_009900775.1 | - |  |
| 20 | intergenic region near integrase | - | - |  |
| 21 | intergenic region | - | (self M120) |  |
| 22 | DNA Polymerase III alpha chain | CEK40467.1 | - |  |
| 23 | putative crossover junction endodeoxyribonuclease | AKP44731.1 | - |  |
| 24 | intergenic region | - | - |  |
| 25 | CRISPR region | - | - |  |
| 26 | - | - | - |  |
| 27 | - | - | - |  |
| 28 | putative phage portal protein | ADK37865.1 | - |  |
| 29 | helix-turn-helix family protein (DNA binding) | WP_021382239.1 | - |  |
| 30 | - | - | - |  |
| 31 | tail fiber protein | CEK40349.1 | CD146 QCD-37x79 QCD-63q42 P7 | 3' ATTTTAGG ATAATAAT 5' |
| 32 | - | - | - |  |
| 33 | - | - | - |  |
| 34 | - | - | - |  |
| 35 | - | - | - |  |
| 36 | - | - | - |  |
| 37 | NUMOD1 domain protein | AJP10597.1 | ATCC43255 DA00305 DA00129 6503 6041 7032994 7032989 NCTC13307 P50 LIBA-5734 LIBA-5719 LIBA-5704 LIBA-5701 ATCC9689 QCD-63q42 pCD630 CD196 | 3' AGAAAAGT TAGACTGT 5' |
| 38 | - | - | - |  |
| 39 | intergenic region | - | T5 | 3' TAAATTTA CAATTTGT 5' |
| CRISPR 5 | |  |  |  |
| 1 | CRISPR region | - | T5 | 3' ATATTTTG GAAATGTA 5' |
| 2 | - | - | - |  |
| 3 | PD-(D/E)XK nuclease superfamily protein | WP_021369992.1 | CD45 CD200 ATCC9689 P50 Y165 | 3' AATTCTAT TCCAGAAA 5' |
| 4 | intergenic region | - | - |  |
| 5 | DNA Polymerase III alpha chain | AKP44758.1 | - |  |
| 6 | hypothetical protein | YP_529607.1 | - |  |
| 7 | putative holin | CEK40597.1 | - |  |
| 8 | putative phage tail tape measure protein | WP_021394514.1 | - |  |
| 9 | intergenic region | - | - |  |
| 10 | - | - | - |  |
| 11 | tail tape measure protein | YP_006990568.1 | - |  |
| 12 | putative major capsid protein A | YP_004306106.1 | - |  |
| 13 | - | - | - |  |
| 14 | putative holin | AKP44718.1 | - |  |
| 15 | hypothetical protein | AKP44743.1 | - |  |
| 16 | glycine rich hypothetical protein | YP_006990577.1 | CD3 LIBA-5701 LIBA-5704 LIBA-5719 LIBA-5734 P50 7032985 7032989 7032994 Y215 | 3' TTGGATTT TCCTGAGA 5' |
| 17 | conserved hypothetical protein | CDS86978.1 | - |  |
| 18 | N-acetylmuramoyl-l-alanine amidase | AKP44625.1 | - |  |
| 19 | intergenic region | - | LIBA-5704 LIBA-5719 | 3' ACAGTTAT ACCTGCAT 5' |
| 20 | putative cell wall hydrolase protein | CDL68832.1 | LIBA-5704 LIBA-5719 7032989 | 3' AAATGAAT ATCCTTAC 5' |
| 21 | tail tape measure protein | YP_006990568.1 | CD175 LIBA-5704 LIBA-5719 BJ08 7032985 M68 CF5 | 3' GTACTTGT TCCGTTCG 5' |
| 22 | conserved hypothetical protein | CEK40753.1 | - |  |
| 23 | intergenic region | - | - |  |
| 24 | putative essential recombination function protein | CDW17246.1 | - |  |
| 25 | putative tail fiber protein | CDU85306.1 | - |  |
| 26 | siphovirus Gp157 family protein | WP_021412133.1 | 840 842 Y165 | 3' CTGACATA TCCTTTAT 5' |
| 27 | conserved hypothetical protein | WP_009894399.1 | BI9 DA00305 CD22 6041 LIBA-5719 QCD-63q42 LIBA-5704 LIBA-5734 LIBA-5701 | 3' GTTACGGT TTTATATT 5' |
| 28 | phage capsid E family protein | WP_021371050.1 | - |  |
| 29 | putative tail fiber protein | YP_009032171.1 | - |  |
| 30 | lambda repressor-like DNA-binding protein | YP_006990520.1 | - |  |
| 31 | - | WP_021359695.1 | - |  |
| 32 | phage terminase large subunit PBSX family | WP_021365843.1 | - |  |
| CRISPR 6 | |  |  |  |
| 1 | antirepressor | - | (self M120) |  |
| 2 | putative phage capsid protein | CDS90461.1 | 7032985 | 3' AAGTGGGT AAATAGTT 5' |
| 3 | putative phage capsid protein | YP_659580.1 | pCD630 | 3' CTGTTGGT TTGAAGCT 5' |
| 4 | pyruvate carboxylase | WP_003421260.1 | (self M120) |  |
| 5 | GIY-YIG catalytic domain protein | WP_021382786.1 | CD160 | 3' ATCTCGGA GATTTTGT 5' |
| 6 | penicillinase repressor family protein | WP_021363140.1 | - |  |
| 7 | - | - | - |  |
| 8 | conserved hypothetical protein | ADK37914.1 | - |  |
| 9 | - | - | - |  |
