# Supplementary material for: Use of single molecule sequencing for comparative genomics of an environmental and a clinical isolate of Clostridium difficile ribotype 078
Source: BMC Genomics. 2016 Dec 13;17:1020. doi: 10.1186/s12864-016-3346-2 (PMC5154133; doi:10.1186/s12864-016-3346-2)
Supplement: Additional file 4: Table S3. — Genome sequences used in this study. (DOCX 13 kb) [file 12864_2016_3346_MOESM4_ESM.docx]

| *C. difficile* phage or strain | | Ribotype | Accession |
| --- | --- | --- | --- |
| ϕC2 | phage | - | NC_009231 |
| phiCDHM1 | phage | - | NC_024144 |
| ΦMMP01 | phage | - | NC_028838 |
| ΦMMP03 | phage | - | NC_028959 |
| ΦCD119 | phage | - | NC_007917 |
| phiCDHM19 | phage | - | NC_028996 |
| ϕCD27 | phage | - | NC_011398 |
| ΦMMP02 | phage | - | NC_019421 |
| ΦCD505 | phage | - | NC_028764 |
| ΦMMP04 | phage | - | NC_019422 |
| phiCDHM13 | phage | - | NC_029116 |
| phiCDHM14 | phage | - | LK985321 |
| ΦCD481-1 | phage | - | NC_028951 |
| ΦCD506 | phage | - | NC_028838 |
| ϕCD38-2 | phage | - | NC_015568 |
| ΦCD111 | phage | - | NC_028838 |
| ΦCD146 | phage | - | NC_028838 |
| ΦCD6356 | phage | - | NC_015262 |
| phiCDHM11 | phage | - | NC_029001 |
| ΦCD24-1 | phage | - | LN681534 |
| CD3 | bacteria | 014 | GCA_000448825.2 |
| CD22 | bacteria | 046 | GCA_000448785.2 |
| CD39 | bacteria | 014-020 | GCA_000448945.2 |
| CD45 | bacteria | 027 | GCA_000449065.2 |
| CD51 | bacteria | 002 | GCA_000449145.2 |
| CD127 | bacteria | 014-020 | GCA_000451325.2 |
| CD160 | bacteria | “Unique” | GCA_000449425.2 |
| CD175 | bacteria | 017 | GCA_000449525.2 |
| CD196 | bacteria | 012 | FN538970.1 |
| CD200 | bacteria | “Unique” | GCA_000449645.2 |
| CD630 | bacteria | 012 | AM180355.1, AM180356.1 |
| CD630DERM | bacteria | 012 | LN614756.1 |
| NCTC 13307 | bacteria | 012 | LN831030.1 |
| QCD-37x79 | bacteria | 027 | GCA_000154685.1 |
| QCD-63q42 | bacteria | 001 | GCA_000154625.1 |
| QCD-97b34 | bacteria | 027 | GCA_000154665.1 |
| ATCC 9689 (CD211) | bacteria | 001 | GCA_000438845.1 |
| LIBA-5701 (NAPCR1) | bacteria | 012 | GCA_000828235.1 |
| LIBA-5704 (NAPCR1) | bacteria | 012 | GCA_000828325.1 |
| LIBA-5719 (NAPCR1) | bacteria | 012 | GCA_000828255.1 |
| LIBA-5734 (NAPCR1) | bacteria | 012 | GCA_000828225.1 |
| LIBA-5784 (NAPCR1) | bacteria | 012 | GCA_000828245.1 |
| P50 | bacteria | SW26 | GCA_000451965.2 |
| BJ08 | bacteria | 017 | CP003939 |
| 7032985 | bacteria | 010 | GCA_000935985.1 |
| 7032989 | bacteria | 010 | GCA_000940315.1 |
| 7032994 | bacteria | 010 | GCA_000939295.1 |
| B11 | bacteria | 027 | FN668941.1, FN668942.1 |
| B19 | bacteria | 027 | FN668944.1 |
| M68 | bacteria | 017 | FN668375 |
| 840 | bacteria | 014-020 | GCA_000449805.2 |
| 842 | bacteria | 014-020 | GCA_000449825.2 |
| 6041 | bacteria | 001 | GCA_000449845.1 |
| 6042 | bacteria | 002 | GCA_000449865.2 |
| 2007855 | bacteria | 027 | FN665654 |
| CF5 | bacteria | 017 | FN665652 |
| CII7xCD13A | bacteria | 039 | GCA_000821005.1 |
| F17xCD13A | bacteria | 039 | GCA_000821025.1 |
| G46 | bacteria | 027 | GCA_000826625.2 |
| DA00193 | bacteria | 053-163 | GCA_000450325.2 |
| DA00305 | bacteria | 001 | GCA_000451345.1 |
| DA00310 | bacteria | 053-163 | GCA_000450685.2 |
| P7 | bacteria | - | GCA_000451605.1 |
| Y165 | bacteria | 014-020 | GCA_000451365.2 |
| Y202 | bacteria | 014-020 | GCA_000451165.2 |
| Y215 | bacteria | SW8 | GCA_000451265.2 |
| Y307 | bacteria | 002 | GCA_000451485.2 |
| R20291 | bacteria | 027 | FN545816.1 |
| ATCC 43255 | bacteria | 087 | GCA_000155025.1 |
| T5 | bacteria | 126 | GCA_001050575.1 |
