# Supplementary material for: Use of single molecule sequencing for comparative genomics of an environmental and a clinical isolate of Clostridium difficile ribotype 078
Source: BMC Genomics. 2016 Dec 13;17:1020. doi: 10.1186/s12864-016-3346-2 (PMC5154133; doi:10.1186/s12864-016-3346-2)
Supplement: Additional file 5: Table S4. — Viral metagenome datasets used for protospacer identification. (DOCX 13 kb) [file 12864_2016_3346_MOESM5_ESM.docx]

| **Dataset** | **Source** |
| --- | --- |
| ICEBERG (all integrated and conjugative elements) | http://db-mml.sjtu.edu.cn/ICEberg/ |
| Phage finder (phantome manually verified prophage predictions) | http://www.phantome.org/Downloads/Prophages/ |
| PhiSpy (phantome manually verified prophage predictions) | http://www.phantome.org/Downloads/Prophages/ |
| EBI phage sequences | http://www.ebi.ac.uk/genomes/phage.html |
| Virsorter Database | http://datadryad.org/resource/doi:10.5061/dryad.b8226/1 |
| 1327 French Lakes_Lake_Bourget | http://metavir-meb.univ-bpclermont.fr/ |
| 1328_French_Lakes_Lake_Pavin |  |
| 1351_Reclaimed_water_Effluent_DNA |  |
| 1469_Human_gut_Subject_1 |  |
| 1470_Human_gut_All_subjects |  |
| 1616_Twins_Feces_Feces_Twins_Reyes |  |
| 1700_Archevir_P5_Ngallou |  |
| 1701_Archevir_P2_Saloon |  |
| 1702_Archevir_P6_Lake_Retba |  |
| 1703_Archevir_P7_Ngallou |  |
| 1704_Archevir_P8_Ngallou |  |
| 1705_Archevir_P9_Ngallou |  |
| 2734_Lakes_AllContigs-VCJUL-1 |  |
| 2735_Lakes_VCJUL-2 |  |
| 2736_Lakes_AllContigs-VCSEP-2A |  |
| 2895_POV_Great_Barrier_Reef_Dunk_Island |  |
| 2896_POV_Great_Barrier_Reef_Fitzroy_Island |  |
| 3506_Anaerobic_digestion_virome_DNAViromeMasterAssembly |  |
| 3816_Marine_Viromes_ALOHA_station_deep_abyss |  |
| 5053_Lough_Neagh_4pW_contigs |  |
| 5062_VirSorter_curated_dataset |  |
| 5127_Far-T4_Lake_Pavin |  |
| 5749_EAfrica_PigFecalvirome_Jamimo_Swine_fecalvirome |  |
| 5754_Marine_Viromes_B47_Bohai_Sea_Sep_2010 |  |
| 5764_EAfrica_PigFecalvirome_Swine_fecalvirome_S23 |  |
| 5967_Biotechnology_Metagenomics_soil |  |
| 6557_EAfrica_PigFecalvirome_S10 |  |
| 6558_EAfrica_PigFecalvirome_S06 |  |
| 6571_Human_Feces_10_eld_contigs |  |
